# P05.26. Systematic review of breastfeeding and herbs

**DOI:** 10.1186/1472-6882-12-S1-P386

**Published:** 2012-06-12

**Authors:** K Budzynska, Z Gardner, J Duguoa, T Low Dog, P Gardiner

**Affiliations:** 1 Boston Medical Center, Boston, USA; 2 Medicinal Plant Program, Dept. of Plant, Soil, and Insect Sciences, Amherst, USA; 3 Toronto Western Hospital, Toronto, Canada; 4 Arizona Center for Integrative Medicine, University of Arizona Health, Tucson, USA

## Purpose

Despite popular and historical use, there has been little modern research conducted to determine the safety and efficacy of herb use during breastfeeding. The purpose of this study was to systematically review the clinical literature on herbal medicine and lactation.

## Methods

Databases such as Pubmed, CAB abstracts, Cochrane clinical trials, HealthStar, CINHAL, and Reprotox were systematically searched for human trials from 1970 till 2010. Reference lists from relevant articles were hand searched.

## Results

Thirty-two studies met the inclusion criteria. Clinical studies were divided into three categories: survey studies (n=11), safety studies (n= 8), and efficacy studies (n= 13). Six studies were randomized controlled trials. The most common herbs studied were St. John’s wort (Hypericum perforatum L.) (n=3), garlic (Allium sativum L.) extract (n=2), and senna (Cassia senna L.) (n=2). Studies were very heterogeneous with regards to study design, herbal intervention, and outcome measures. Overall, poor methodological quality predominated among the studies.

## Conclusion

Our review concludes that further research is needed to assess the prevalence, efficacy, and safety of herbs during breastfeeding.

